# Supplementary figures and images for: High Serum Levels of IL-6 Predict Poor Responses in Patients Treated with Pembrolizumab plus Axitinib for Advanced Renal Cell Carcinoma
Source: Cancers (Basel). 2022 Dec 3;14(23):5985. doi: 10.3390/cancers14235985 (PMC9738341; doi:10.3390/cancers14235985)

Figure S1

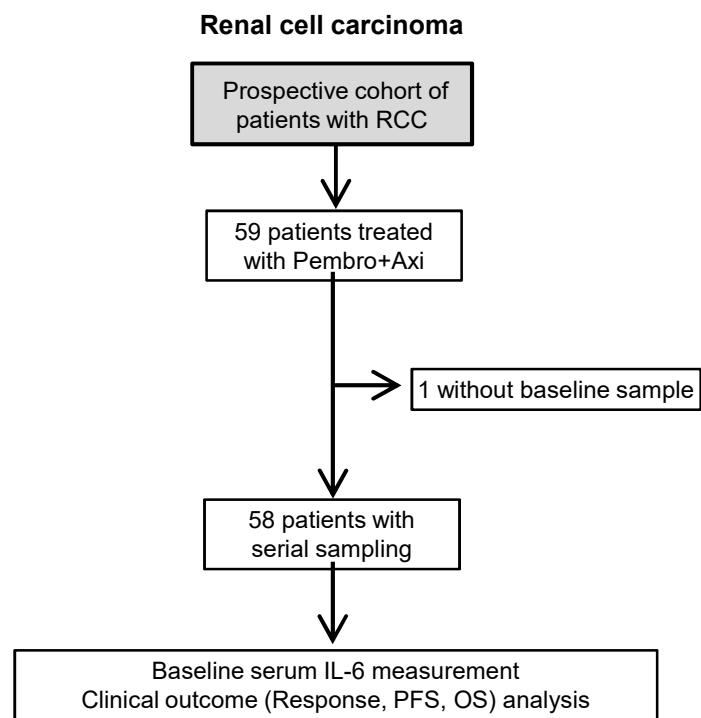

Supplement: Supplementary file 1 [file cancers-14-05985-s001.zip › cancers-2035420-supplementary.pdf]
